# Differential gene expression mediated by 15-hydroxyeicosatetraenoic acid in LPS-stimulated RAW 264.7 cells

**DOI:** 10.1186/1475-2875-8-195

**Published:** 2009-08-11

**Authors:** Alexandra C Schrimpe, David W Wright

**Affiliations:** 1 Department of Chemistry, Vanderbilt University, Nashville, Tennessee 37235, USA

## Abstract

**Background:**

Given the immuno-modulatory activity of native haemozoin (Hz), the effects of constitutive Hz components on immune response are of interest. Recently, gene expression changes mediated by HNE and the synthetic analogue of Hz, beta-haematin (BH), were identified and implicated a significant role for lipid peroxidation products in Hz's activity. The study presented herein examines gene expression changes in response to 15(S)-hydroxyeicosatetraenoic acid (HETE) in a model macrophage cell line.

**Methods:**

LPS-stimulated RAW 264.7 macrophage-like cells were treated with 40 μM 15(S)-HETE for 24 h, and microarray analysis was used to identify global gene expression alterations. Fold changes were calculated relative to LPS-stimulated cells and those genes altered at least 1.8-fold (*p *value ≤ 0.025) were considered to be differentially expressed. Expression levels of a subset of genes were assessed by qRT-PCR and used to confirm the microarray results.

**Results:**

Network analysis revealed that altered genes were primarily associated with "lipid metabolism" and "small molecule biochemistry". While several genes associated with PPAR-gamma receptor-mediated signaling were differentially expressed, a number of genes indicated the activation of secondary signaling cascades. Genes related to cytoadherence (cell-cell and cell-matrix), leukocyte extravasation, and inflammatory response were also differentially regulated by treatment, supporting a potential role for 15(S)-HETE in malaria pathogenesis.

**Conclusion:**

These results add insight and detail to 15-HETE's effects on gene expression in macrophage-like cells. Data indicate that while 15-HETE exerts biological activity and may participate in Hz-mediated immuno-modulation, the gene expression changes are modest relative to those altered by the lipid peroxidation product HNE.

## Background

Although haem is a vital cofactor for a diverse set of proteins involved in respiration, oxygen transport, and drug detoxification, the accumulation of free haem has deleterious effects. Haem is capable of binding to lipid bilayers, catalyzing lipid peroxidation, inhibiting enzymatic activity, and lysing cells and parasites [1,2]. Many organisms utilize the haem oxygenase pathway to degrade free haem. Blood-feeding *Plasmodium *parasites, the source of malaria infection, lack such a pathway. Consequently, haem released during haemoglobin catabolism is sequestered as the insoluble crystalline "malaria pigment" (i.e., haemozoin [Hz]). As most of the haem is occluded within the crystal, the parasite is protected.

Hz is composed of five-coordinate Fe (III) protoporphyrin IX dimers covalently bound by reciprocal iron-carboxylate bonds [3]. The remaining propionate side chains of adjacent dimers form hydrogen bonds, resulting in an extended dimeric network producing the Hz crystal. In its native state, Hz is coated by an array of host- and parasite-derived lipids, proteins, and nucleic acids [4]. Analysis of the lipid component identified peroxidation products including a racemic mixture of 5-, 8-, 9-, 11-, 12-, and 15-hydroxyeicosatetraenoic acids (HETEs) and 9- and 13- hydroxyoctadecadienoic acids (HODEs) [5]. Elevated levels of 4-hydroxynonenal (HNE) were also detected in haemozoin-laden monocytes [6] at the highest reported concentration of any biological system to date [7].

Rupture of parasitized red blood cells (RBCs) releases cellular debris, including residual bodies containing Hz, into the host's vasculature and triggers an innate immune response. The typical response of phagocytic cells to such foreign material includes oxidative burst and rephagocytosis, however, phagocytosis of Hz impairs these innate functions [8-10]. It has been suggested that Hz's immunological activity may not stem from the haem moiety but from nonspecific toxins [11], such as lipid peroxidation products, present on its surface and introduced into the cell during phagocytosis

The cellular response to several lipid peroxidation species associated with Hz is well documented and indicates an involvement in malaria pathophysiology. Recently, two components of native Hz were targeted as potential players involved in macrophage dysfunction [12]. Microarray analysis of the response to HNE and Hz's biologically naïve synthetic analogue, β-haematin (BH), indicated a potential role for HNE in malaria pathogenesis. It seemed probable, given HNE-mediated gene expression changes, that other biologically active lipid peroxidation products generated by Hz, including 15-HETE, may be active in the disease's pathogenesis.

Macrophage-like cells treated with 15-HETE exhibited impaired PMA-activated NADPH oxidase and LPS-stimulated inducible nitric oxide synthase (iNOS) activities, mimicking Hz-mediated monocyte immunomodulation [13]. 15-HETE was also reported to enhance vascular permeability/oedema [14] and RBC adherence to endothelia [15], two hallmarks of malarial infection. The present study examined steady-state gene expression changes induced by 15-HETE in activated RAW 264.7 model macrophage cells in the context of a nonspecific malaria toxin that may be involved in disease pathophysiology.

## Methods

### Cell culture

Murine macrophage-like RAW 264.7 cells (American Type Culture Collection TIB-71, Monassas, VA) were cultured under standard incubation conditions (37°C, 5% CO_2_) and grown in RPMI supplemented with 10% FBS (Atlanta Biologicals, Atlanta, GA) and 1 μg/mL P/S (Cellgro MediaTech, Herndon, VA). Cells were plated at a density of 4 × 10^6 ^cells/well in six well plates and incubated for 24 h prior to treatment.

### Cell treatment and RNA isolation

Cells were washed once with Dulbecco's PBS (DPBS) and treated with 40 μM 15(S)-HETE. Immediately following treatment, LPS was added to all wells at a final concentration of 1 μg/mL. After 24 h, cells were washed three times with DPBS and scraped from the wells. Three biological replicates (composed of six pooled wells each) per sample were prepared. Total RNA was isolated using the Versagene RNA purification and DNase treatment kits, following the manufacturer's recommendations.

### Microarray analysis

Microarray analysis was performed by the Vanderbilt Microarray Shared Resource. Three biological replicates of each treatment were analyzed for quality (Agilent 2100 Bioanalyzer, Agilent Technologies, Palo Alto, CA). One microgram of total RNA (30 ng mRNA) was used to generate first strand cDNA using the NanoAmp RT-IVT labeling kit according to the manufacturer's protocol. Following first strand synthesis, second strand synthesis was completed. The resulting cDNA was then purified using an ABI kit-provided column and the entire reaction was used in an IVT reaction to generate DIG-labeled cRNA. The cRNA was then purified using a kit-provided column and assessed for quality on an Agilent Bioanalyzer. All reactions meeting ABI criteria in terms of quantity and size of target produced were fragmented and then hybridized to an ABI mouse genome survey microarray for 16 h with agitation at 55°C per the manufacturer's protocol. Following the addition of the chemiluminescence reaction substrate, each array was immediately imaged on the 1700 Chemiluminescent Analyzer, and a primary analysis was completed by the AB1700 Expression Array System Software (v 1.1.1). Expression values were quantile normalized and filtered based on S/N (> 3) and flag value (< 5000). GeneSpring GX 7.3.1 software (Agilent Silicon Genetics, Redwood City, CA) was used to determine statistically significant differentially expressed genes. T-tests were performed on probes altered by ≥ 1.8-fold in 2 of 3 samples (0.025 *p *value cut-off, Benjamini-Hochberg multiple testing correction, parametric test, variances not assumed equal) in treated stimulated cells (experimental) relative to stimulated cells (control). Genes were classified according to genes ontology (GO) terms using GeneSpring. In accordance with MIAME procedure, microarray data have been submitted to the NCBI Gene Expression Omnibus and can be found under series number GSE15070. Nomenclature for genes and proteins is as described by the Mouse Genome Informatics (MGI) database guidelines.

### Ingenuity pathway analysis

Ingenuity Pathways Analysis (IPA) was used for gene expression analysis (Ingenuity Systems^®^). A data set containing gene identifiers and corresponding expression values was uploaded into the application, and each identifier was mapped to its corresponding gene object in the Ingenuity knowledge base (IKB). A functional analysis was performed to determine biological functions that were most significant to the genes in the data set. A network analysis was also performed whereby focus genes were overlaid onto a global molecular network developed from information contained in the IKB. Networks of focus genes were then algorithmically generated based on their connectivity. A functional analysis of each network identified the biological functions that were most significant to the genes in the network, and canonical pathway analysis identified the pathways from the IPA library of canonical pathways that were most significant to the data set. Fischer's exact test was used to calculate a *p *value determining the probability that that each biological function assigned to a network or data set, or the association between the genes in the data set and the canonical pathway, are explained by chance alone.

### Real-time reverse transcription polymerase chain reaction

Quantitative real-time reverse transcription polymerase chain reaction (qRT-PCR) was used to validate the expression levels of genes identified as differentially expressed by microarray analysis. Quadruplicate measurements for n = 3 independent biological replicates per sample were performed. cDNA was reverse-transcribed from 0.5 μg of total RNA using random hexamer primers and Superscript II Reverse Transcriptase (Invitrogen). Reactions were purified using Qiagen's PCR Purification Kit following the manufacturer's protocol. Following RT, all assays were performed with Applied Biosystems TaqMan FAM labeled 20× probes: *Arf3 *(Taqman assay Mm00500194_m1), *Cldn11 *(Mm00500915_m1), *Cxcl11 *(Mm00444662_m1), *Mapk14 *(Mm00442497_m1), *Prdx1 *(Mm01621996_s1), *Sdc1 *(Mm00448918_m1), and *Egr1 *(Mm00656724_m1). Ywhaz was chosen as the endogenous control based on results obtained from an Applied Biosystems mouse endogenous control array. cDNA amplification was performed using TaqMan 2× Universal PCR Master Mix (Applied Biosystems), and standard Taqman cycling conditions were used as specified by the manufacturer. Cycling and data collection were performed using the Applied Biosystems 7900 HT instrument, and analysis was performed using SDS software to calculate Ct values for each detector. Ct values were processed based on the comparative Ct method where the relative transcript level of each target gene was calculated according to the equation 2^-ΔCt^, where ΔCt is defined as Ct target gene – Ct *Ywhaz*.

## Results

### Functional analysis of gene expression changes induced by 15(S)-HETE

**Figure 1:**
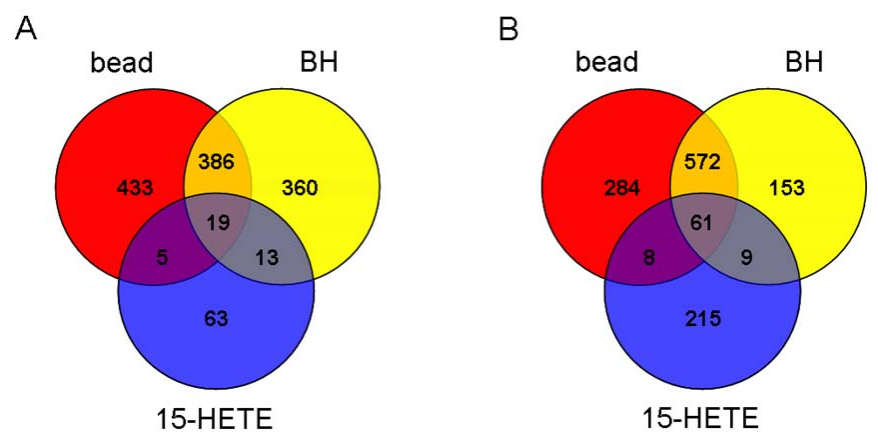
**Comparison of differentially expressed genes mediated by 15-HETE, beta-haematin, and latex beads**. Venn diagrams show the intersection of genes that were transcriptionally altered by 40 μM 15-HETE with those altered by latex bead treatment and serum-opsonized beta haematin (BH) (0.1 mg/mL). Numbers represent statistically significant (*p *≤ 0.025) transcripts up- or down-regulated ≥ 1.8-fold in 2 of 3 samples, relative to LPS-stimulated untreated cells at 24 h. (A) Decreased and (B) increased expression are shown separately.

LPS-stimulated macrophage-like RAW 264.7 cells were treated for 24 h with 40 μM 15(S)-HETE based on the estimate that trophozoites and Hz contained 33–39 μmol 15-HETE/L RBC [5]. Statistically significant (*p *≤ 0.025) changes in gene expression (fold change ≥ 1.8 relative to stimulated cells) were identified by microarray analysis. Given that this study aims to explore potential alterations in gene expression that are incurred by 15-HETE during haemozoin phagocytosis, differentially expressed mRNAs were controlled by comparison with a particulate latex bead challenge and BH treatment under the same conditions. Figure 1 illustrates that 15-HETE had a much greater effect on induction of gene expression than repression (293 transcripts versus 100 transcripts, respectively), but overall was very modest compared to either latex bead or BH treatment.

Ingenuity Pathway Analysis (IPA) software was used to examine biological relationships associated with 15-HETE-mediated expression changes. Identifiers and relative levels of altered genes comprising the data set were imported and mapped by IPA for comparison to molecules within the Ingenuity knowledge base (IKB). Two types of IPA analyses were performed. First, a network analysis was employed to reveal direct and indirect relationships that exist between specific genes in the data set. This analysis resulted in the generation of a network map which illustrates direct and indirect connections between focus genes. Second, a functional analysis was performed to identify the biological processes that are most relevant to the entire set of differentially expressed genes. This analysis resulted in a list of significant biological functions associated with the data set as a whole. Functional analyses were also used to find biological processes associated with individual networks. Focus genes, imported genes that are eligible for generating interaction networks based on incorporation in IKB, were used to identify relationships based on known interactions in the literature. Each network is associated with a score indicating the likelihood that the focus genes occur in the network by random chance. Networks scoring 10 or higher (score is defined as -log (*p *value)) are considered significant.

**Figure 2:**
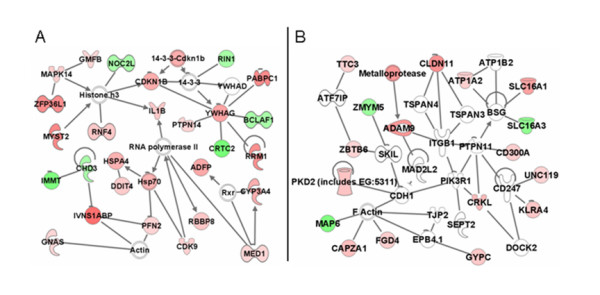
**Ingenuity Pathway network analysis**. Transcripts altered ≥ 1.8-fold (*p *≤ 0.025) in 15-HETE-treated RAW 264.7 cells (i.e., focus genes) were overlaid onto a global molecular network developed from information contained in the Ingenuity knowledge base (IKB). Networks of these focus genes were algorithmically generated based on their connectivity. Networks show direct interactions between focus genes altered by 15-HETE treatment and associated molecules within the IKB. Genes or gene products are represented as nodes, and the biological relationship between two nodes is represented as an edge (line). White nodes represent IKB molecules that are associated with focus genes. All edges are supported by at least one reference stored in the IKB. The intensity of node color indicates the degree of up- (red) or down- (green) regulation. Networks reflect (A) lipid metabolism and small molecule biochemistry and (B) molecular transport and cellular movement.

Among the transcripts modulated by 15-HETE treatment, 263 were eligible for network analysis based on IPA criteria, mapping to 11 relevant interaction networks. The most significant network (Figure 2a) had a score of 51 and associated 27 focus genes. Several transcriptional regulators were among the products encoded by these genes (*Bclaf1*, *Med1*, *Noc2l*, *Rnf4*, and *Zfp36l1*). This network also contained *Il1b*, *Cyp3a4*, *Gnas*, and *Adfp*. A functional analysis performed on this particular network indicated that the differentially expressed genes were associated with "lipid metabolism" and "small molecule biochemistry" (p = 1.27 × 10^-4^).

Figure 2b shows the second most significant interaction network identified by IPA network analysis. Eighteen focus genes were incorporated into the network with a score of 29. Functional analysis of the network revealed that the genes were involved in "molecular transport" (p = 9.42 × 10^-7^) and "cellular movement" (p = 9.77 × 10^-6^). This network is enriched with focus genes encoding molecules associated with the plasma membrane such as *Pkd2*, *Cd300a*, *Cldn11*, *Gypc*, *Klra4*, peptidase *Adam9 *and transporters *Atp1a2*, *Slc16a1*, and *Slc16a3*. Consistent with these genes, the network predicted interactions with several other plasma membrane molecules (*Tjp2*, *Bsg*, *Cdh1*, *Tspan3*, *Tspan4*, *Cd247*, *Itgb1*, and *Atp1b2*) that were not present in the data file.

### Molecular and cellular functions controlled by 15(S)-HETE

**Figure 3:**
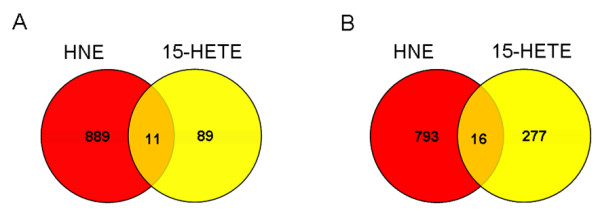
**Comparison of differentially expressed genes mediated by 15-HETE and HNE**. Data sets for each treatment group (15-HETE or HNE) were generated from statistically significant (*p *≤ 0.025) transcripts up- or down-regulated ≥ 1.8-fold in 2 of 3 samples relative to untreated LPS-stimulated cells. Venn diagrams show intersections of the resulting 15-HETE and HNE data subsets. (A) Decreased and (B) increased expression are shown separately.

It is thought that Hz impairs cellular function through the generation and introduction of toxic species such as lipid peroxidation products into cells. Previously, the ability of HNE to stimulate a transcriptional response was examined in macrophage-like cells [12]. It was observed that HNE significantly impacted a wide range of steady-state responses (e.g., macrophage activation, immune and inflammatory responses, NF-κB signal transduction, ECM degradation, and dyserythropoiesis). Comparison of the number of gene expression changes influenced by 15-HETE and HNE indicates that 15-HETE modulates a number of mRNA targets but is a much less potent agent than HNE (Figure 3).

IPA was also used to perform a functional analysis on genes within the entire data set. Comparison of the biological functions modulated by 15-HETE (Table 1) and HNE [12] revealed that 15-HETE affected a considerably smaller group of transcripts than HNE but mediated a comparable response in terms of the number of molecular and cellular functions and the specific categories affected. Both lipid peroxidation products altered "Cell Cycle", "Cell Morphology", "Cellular Assembly and Organization", "Cell Death", "Cellular Development", "Cell Growth and Proliferation", "Gene Expression", and "Small Molecule Biochemistry". 15-HETE affected several unique categories including "Carbohydrate Metabolism", "Drug Metabolism", "Lipid Metabolism", "Molecular Transport", "RNA Damage and Repair", and "RNA Post-Translational Modification".

**Table 1:** Functional Analysis of 15-S-HETE Data Set^*a*^

Biological Function	*p *value
carbohydrate metabolism	6.42 ×10^-6^
cell cycle	1.28 × 10^-4^
cell death	1.93 × 10^-5^
cell morphology	2.53 × 10^-4^
cellular assembly and organization	5.30 × 10^-4^
cellular compromise	4.52 × 10^-4^
cellular development	3.42 × 10^-5^
cellular growth and proliferation	3.81 × 10^-4^
drug metabolism	3.81 × 10^-4^
gene expression	2.22 × 10^-4^
lipid metabolism	1.25 × 10^-3^
molecular transport	3.81 × 10^-4^
RNA damage and repair	3.79 × 10^-4^
RNA post-transcriptional modification	3.79 × 10^-4^
small molecule biochemistry	3.81 × 10^-4^

^*a *^Ingenuity Pathway Analysis uses a right-tailed Fisher exact test to calculate *p *values. Significance values for each data set indicate the probability that the association between the genes and the given biological function is due to random chance.

Both IPA network and functional analyses identified a large group of "lipid metabolism" and "carbohydrate metabolism" expression changes. Given that *Il1b *acts upstream of *Cyp3a4*, *Ugdh*, *Gnas*, *Gm2a*, *Psen1*, and *Il15*, stimulated *Il1b *expression may be indirectly involved in the up-regulation of each of these genes in this study. Expression of several "small molecule biochemistry" transcriptional regulators (*Bclaf1, Med1, Rnf4, Noc2l*, and *Zfp36l1*) was also identified.

### Differential gene expression in the context of malaria pathogenesis

Differentially expressed genes were sorted into lists based on the direction of regulation, and corresponding Gene Ontology (GO) categories were identified. Gene expression alterations mediated by 15-HETE were compared to two groups of transcripts. The first group consisted of specific genes or gene products associated with human [16] or murine [17,18] models of malarial infection or Hz exposure [19]. Common transcripts were primarily associated with "cell-to-cell signaling and interaction" and "immune response" (e.g., *Fcgrt*, *Cd86*, *C5ar1*, *Ccr4*, *Mapk14*, *Pik3ap1*, *Tapbp*, and *Tnfaip6*). Enhanced expression of guanylate nucleotide binding proteins (*Gbp*) 1 and 3 observed in this study was consistent with expression changes reported in human and experimental murine malaria [16-18,20]. The second group included genes classified under specific GO processes that are overexpressed in the *Plasmodium yoelii *model [20] and/or naturally acquired *Plasmodium falciparum *infections [21], including cell-cell signaling, defense response, immune response, inflammatory response, and signal transduction, among others. Differential expression mediated by 15-HETE treatment that correlated with either of the two groups described above is listed in Table 2. The relatively limited correlation reflects differences between 15-HETE-mediated expression changes in this model and expression changes observed during naturally acquired or experimental malaria. While RAW 264.7 cells have previously been shown to mimic monocyte/macrophage immuno-modulation in the presence of HNE [12], the findings presented herein suggest that 15-HETE is not a major contributor to the altered immune response observed in these cells types during infection.

**Table 2:** Select Gene Expression Changes Mediated by 15-HETE^*a*^

Gene Symbol	Fold Change	Probe ID	Description	Entrez ID	Ref

Electron Transport [21]					

Cyp3a11	3.6	516253	Cytochrome P450, family 3, subfamily a, polypeptide 11	13112	
Smox	5.1	560410	Spermine oxidase	228608	
Ugdh	1.9	500013	UDP-glucose dehydrogenase	22235	

Regulation of Transcription, DNA-Dependent [21]					

Bclaf1	-1.9	549609	BCL2-associated transcription factor 1	72567	
Cdk9	2.1	392872	Cyclin-dependent kinase 9 (CDC2-related kinase)	107951	
Creg1	1.9	760346	Cellular repressor of E1A-stimulated genes 1	433375	
Egr1	-4.7	524988	Early growth response 1	13653	
Fbxl11	-2.9	464056	F-box and leucine-rich repeat protein 11	225876	
Fli1	2.3	407869	Friend leukemia integration 1	14247	
Fliih	4.6	706377	Flightless I homologue (*Drosophila*)	14248	
Hlx1	2.1	915372	H2.0-like homeo box 1 (*Drosophila*)	15284	
Mxd1	2.1	520449	MAX dimerization protein 1	17119	
Myst2	5.4	494053	MYST histone acetyltransferase 2	217127	
Pou2f2	-4.4	911620	POU domain, class 2, transcription factor 2	18987	
Pparbp	1.9	553770	Peroxisome proliferator activated receptor binding protein	19014	
Pspc1	2.0	474771	Paraspeckle protein 1	66645	
Rab11a	2.0	359489	RAB11a, member RAS oncogene family	53869	
Rnf4	2.5	567180	Ring finger protein 4	19822	
Tsc22d3	2.0	700170	TSC22 domain family 3	14605	
Zfp482	2.0	435236	Zinc finger protein 482	241322	

Protein Biosynthesis [20,21]					

Eprs	4.9	455664	Glutamyl-prolyl-tRNA synthetase	107508	

Protein Folding [21]					

Clpx	2.4	733670	Caseinolytic peptidase X (*E. coli*)	270166	
Hspa4	3.5	578003	Heat shock protein 4	15525	

Ubiquitin Cycle [21]					

Cul7	0.4	742757	Cullin 7	66515	
Fbxo3	1.9	832607	F-box only protein 3	57443	
Ube2l6	2.0	401185	Ubiquitin-conjugating enzyme E2L 6	56791	[16]

Intracellular Protein Transport [21]					

Ap1s2	2.0	605927	Adaptor-related protein complex 1, sigma 2 subunit	108012	
Arf3	4.4	652348	ADP-ribosylation factor 3	11842	
Sort1	2.4	339169	Sortilin 1	20661	

Response to Stress [21]					

Mapk14	2.2	755610	Mitogen activated protein kinase 14	26416	[16]
Prdx1	2.1	530413	Peroxiredoxin 1	18477	[17]
Prdx6	-2.0	825043	Peroxiredoxin 6	11758	

Defense Response [20,21]					

Bst1	2.1	837914	Bone marrow stromal cell antigen 1	12182	
Klra18	2.5	806675	Killer cell lectin-like receptor, subfamily A, member 18	93970	
Tapbp	2.0	928057	TAP binding protein	21356	[20]

Inflammatory Response [20,21]					

Abcb1a	2.6	677412	ATP binding cassette, sub-family B (MDR/TAP), member 1A	97570	
Ca2	4.0	574832	Carbonic anhydrase 2	88269	
Card12	2.2	336709	Caspase recruitment domain family, member 12	268973	
Cdkn1b	3.7	516253	Cytochrome P450, family 3, subfamily a, polypeptide 11	104565	
Clu	4.0	379462	Clusterin	88423	
Cr1l	1.9	538208	Complement component (3b/4b) receptor 1-like	88513	
Cyp3a11	3.7	516253	Cytochrome P450, family 3, subfamily a, polypeptide 11	88609	
Fyn	2.50	766362	Fyn proto-oncogene	95602	
H2-Q8	1.8	712519	Histocompatibility 2, Q region locus 8	95937	
Hnrnpa3	4.8	903894	Heterogeneous nuclear ribonucleoprotein A3	1917171	
Mrc1	2.9	331550	Mannose receptor, C type 1	97142	
Pole4	2.3	508321	Polymerase (DNA-directed), ε 4 (p12 subunit)	1914229	
Ppp3r1	2.7	716541	Protein phospatase 3, regulatory subunit B, α isoform (calcineurin B, type I)	107172	
Procr	2.0	431405	Protein C receptor, endothelial	104596	
Rrm1	5.6	865694	Ribonucleotide reductase M1	98180	
Serpinb2	4.0	860577	Serine (or cysteine) proteinase inhibitor, clade B, member 2	97609	

Leukocyte Extravasation and Signalling					

Arhgap12	2.2	465731	ρ GTPase activating protein 12	1922665	
Crkl	3.0	389169	V-crk sarcoma virus CT10 oncogene homologue (avian)-like	104686	
Ptk2b	2.6	867483	PTK2 protein tyrosine kinase 2 β	104908	

Immune Response [20,21]					

Cxcl11	5.1	921243	Chemokine (C-X-C motif) ligand 11	56066	
Ddx58	7.1	438990	DEAD (Asp-Glu-Ala-Asp) box polypeptide 58	230073	
Fcgrt	2.0	390657	Fc receptor, IgG, α chain transporter	14132	[16]
Gbp1	2.9	586296	Guanylate nucleotide binding protein 1	14468	[16,18,20]
Gbp3	3.0	405120	Guanylate nucleotide binding protein 3	55932	[17,20]
Ifit3	3.2	888038	Interferon-induced protein with tetratricopeptide repeats 3	15959	
Il1a	5.1	595893	Interleukin 1 α	96542	
Il1b	2.4	734612	Interleukin 1 β	16176	
Il15	1.9	876196	Interleukin 15	16168	

Cell Cycle [20]					

Ccnf	2.4	767163	Cyclin F	12449	
Cdkn1b	3.7	704876	Cyclin-dependent kinase inhibitor 1B (P27)	12576	
Pmp22	1.9	616997	Peripheral myelin protein	18858	
Rhob	-2.2	925472	Ras homologue gene family, member B	11852	

Cell Adhesion [21]					

Cldn11	4.9	338333	Claudin 11	18417	
Scarb2	2.5	561450	Scavenger receptor class B, member 2	12492	
Tnfaip6	1.9	614886	Tumor necrosis factor alpha induced protein 6	21930	[16]

Signal Transduction [20]					

Ccr4	2.2	618105	Chemokine (C-C motif) receptor 4	12773	[18]
Cd86	3.2	908805	Cd86 antigen	12524	[20,22]
Gnas	1.8	646267	GNAS (guanine nucleotide binding protein, alpha stimulating) complex locus	14683	
Ms4a4c	**2.8**	791872 495283	Membrane-spanning 4-domains, subfamily A, member 4C	64380	
Olfr472	-2.2	591718	Olfactory receptor 472	258770	
Prkrir	3.4	561755	Protein-kinase, interferon-inducible double stranded RNA dependent inhibitor, repressor of (P58 repressor)	72981	
Ptger2	3.0	912597	Prostaglandin E receptor 2 (subtype EP2)	19217	
Rin1	-1.9	478326	Ras and Rab interactor 1	225870	
Ywhag	4.1	606287	3-monooxygenase/tryptophan 5-monooxygenase activation protein, gamma polypeptide	22628	

G-Protein Coupled Receptor Protein Signaling Pathway [21]					

Olfr1303	-2.0	366625	Olfactory receptor 1303	258397	
Olfr316	-1.9	903210	Olfactory receptor 316	258064	
Olfr435	-3.2	810459	Olfactory receptor 435	258647	
Slc19a2	1.9	763767	Solute carrier family 19 (thiamine transporter), member 2	116914	

Cell-Cell Signaling [21]					

Wnt6	2.0	590115	Wingless-related MMTV integration site 6	22420	

Development [21]					

Egfl4	-2.2	914308	EGF-like-domain, multiple 4	269878	
Lrp6	-2.3	691244	Low density lipoprotein receptor-related protein 6	16974	
Pgf	2.9	932795	Placental growth factor	18654	

Metabolism [20]					

Atp1a2	2.1	684165	ATPase, Na+/K+ transporting, α 2 polypeptide	98660	
Atp2c1	2.0	388850	ATPase, Ca^2+ ^sequestering	235574	
Echdc3	2.2	331450	Enoyl Coenzyme A hydratase domain containing 3	67856	
Hsd17b4	2.2	303973	Hydroxysteroid (17-beta) dehydrogenase 4	15488	
Mmp9	-12.3	710293	Matrix metalloproteinase 9	17395	
Oas3	-3.0	487213	2'-5' oligoadenylate synthetase 3	246727	[16]

Carbohydrate Transport [21]					

Slc35a4	2.5	318829	Solute carrier family 35, member A4	67843	

Protein Transport [21]					

Exoc2	3.0	498825	Exocyst complex component 2	66482	
Nupl2	2.1	868036	Nucleoporin like 2	231042	
Rab20	2.0	410549	RAB20, member RAS oncogene family	19332	
Rap2b	2.2	471908	RAP2B, member of RAS oncogene family	74012	
Rheb	1.9	653270	RAS-homologue enriched in brain	19744	
Zfyve20	2.9	669220	Zinc finger, FYVE domain containing 20	78287	

Protein Ubiquitination [21]					

Trim12	2.7	454451	Tripartite motif protein 12	76681	
Trim34	2.9	600486	Tripartite motif protein 34	94094	

Differentiation [21]					

Ndrg2	2.1	468211	N-myc downstream regulated gene 2	29811	

Other					

Pik3ap1	2.0	646764	Phosphoinositide-3-kinase adaptor protein 1	83490	[16]

^*a *^Transcripts altered ≥ 1.8-fold (*p *≤ 0.025) in 15(S)-HETE-treated RAW 264.7 cells that are associated with specific genes or gene products correlated to malaria (referenced in column 6) or genes that are classified with specific over-expressed GO biological processes in malaria models (referenced with biological process heading), are shown in the table. Fold changes (FC) represent the average of three independent biological experiments. **Bold **FC indicate that multiple probes gave analogous results (average FC is shown).

### Validation of microarray results

**Figure 4:**
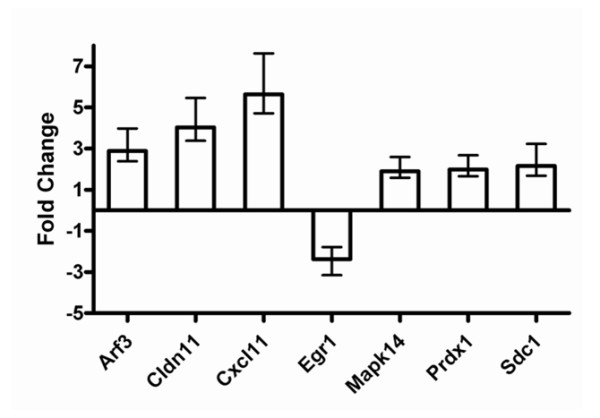
**Quantitative real-time RT-PCR validation of microarray results**. RAW 264.7 cells were stimulated with 0.1 μg/mL LPS and treated with 40 μM 15-HETE for 24 h prior to RNA extraction. Fold-changes (treated stimulated cells relative to stimulated cells) are shown ( ± 99% confidence interval for quadruplicate measurements of n = 3 biological replicates). Abbreviations: *Arf3 *(ADP-ribosylation factor 3), *Cldn11 *(claudin 11), *Cxcl11 *(chemokine (C-X-C motif) ligand 11), *Egr1 *(early growth response 1), *Mapk14 *(mitogen-activated protein kinase 14), *Prdx1 *(peroxiredoxin 1), and *Sdc1 *(syndecan 1).

qRT-PCR was used to confirm several genes susceptible to differential regulation by 15-HETE. The analysis focused on selected genes implicated in the host response to malaria. The results shown in Figure 4 are expressed as fold change relative to LPS-stimulated cells. In agreement with the microarray results in terms of magnitude and direction of change, 15-HETE stimulated the expression of *Arf3 *(ADP-ribosylation factor 3), *Cldn11 *(claudin 11), *Cxcl11 *(chemokine (C-X-C motif) ligand 11), *Mapk14 *(mitogen-activated protein kinase 14), *Prdx1 *(peroxiredoxin 1), and *Sdc1 *(syndecan 1) and repressed the expression of *Egr1 *(early growth response 1).

## Discussion

Global responses to malaria infection have been examined at the molecular level in the blood of human victims [16,21], malaria positive tissue [22], and both murine [17,18,20] and monkey [23] malaria models using microarray technology. Perturbations of gene expression associated with erythropoiesis, glycolysis, metabolism, B-cell activation, and inflammation were frequently identified in these analyses; however, the specific agents responsible for mediating expression changes remain unknown. Accumulating evidence supports the hypothesis that many of the adverse effects of malaria are not caused directly by the parasite, but by endogenous toxins generated during interactions with parasite-derived species such as Hz [24].

The immuno-modulatory response to native Hz has been recapitulated using individual components of Hz (i.e., membrane lipids from erythrocyte ghosts incubated with BH) in a model system [13]. Macrophage-like cells treated with the reaction supernatant exhibited a dose-dependent impairment of PMA-activated NADPH oxidase and LPS-stimulated iNOS activities. Neither BH- nor ghost-supernatant alone altered NADPH or iNOS activity, indicating that lipid peroxidation products generated during reactions between BH and ghost membranes were responsible for the inhibitory effects. Several laboratories have shown that biologically relevant levels of the individual lipid peroxidation products HNE and 15-HETE were capable of mimicking the dysfunctional response to Hz phagocytosis, suggesting the basis of Hz activity [13,25,26].

Considering these results, global responses to individual Hz constituents are of particular interest. The ability of specific and nonspecific malaria toxins to stimulate changes in gene expression has recently been examined in macrophage-like cells [12]. Microarray analyses of two individual Hz components (i.e., BH and HNE) indicated that while BH primarily elicited a phagocytic response, HNE significantly perturbed a myriad of biological processes. These results substantiated further exploration of a potential role for the Hz-associated lipid peroxidation product 15-HETE.

In the current study, activated RAW 264.7 macrophage-like cells were treated with 15-HETE, and mRNA levels were assessed at 24 h to mimic a steady-state response that would be relevant to an established malaria infection. IPA software was utilized to perform complementary network (Figure 2) and functional (Table 1) analyses for identification of biological relationships within the data. Figure 3 shows that the number of expression changes mediated by 15-HETE was modest relative to the previously reported global response to HNE [12]. Unlike the mode of action behind HNE's biological activity (forming adducts to cellular nucleophiles and subsequently modulating intracellular signaling), 15-HETE serves as a ligand for the nuclear PPARγ receptor [27]. As expected, downstream PPARγ signaling transcripts (e.g., *Adfp*, *Ca2*, *Cyp3a4*, *M6pr*, *M6prbp1*, *Med1*, *Med7*, and *Sdc1*) were elevated in response to 15-HETE.

### Cytoadherence

A balance between removal of *Plasmodium *from circulation and sequestration inside host cells is crucial for parasite survival during infection. Sequestration is mediated by cytoadherence, specifically, the adherence of parasitized RBCs (PRBCs) and leukocytes to capillary and post-capillary venular endothelial cells (EC). This cytoadherence reduces blood flow and causes metabolic dysfunction [28] and is thought to be a major factor associated with cerebral malaria (CM). The mechanism(s) used for adhesion and migration involve the expression of constitutive ligands (i.e., adhesion molecules) and receptors on PRBCs or leukocytes and EC. Cell-cell and cell-matrix interactions are also mediated by the secretion of microbial products or cytokines, which enhance the expression of inducible adhesion molecules.

Investigation of potential arachidonic acid metabolite involvement in cytoadherence identified 15-HETE as an agent capable of stimulating basal adhesion of erythrocytes [15] and monocytes to EC [29,30]. In this study, 15-HETE induced the expression of several transcripts involved in integrin signaling (e.g., *Crkl*, *Rap2b*, *Arf3*). The expression of genes encoding *Pkd2 *and *Sdc1*, which are involved in cell-cell and cell-matrix interactions, and *Ptpn14*, which has alleged involvement in cell adhesion, were also induced by 15-HETE.

### Leukocyte extravasation and chemotaxis

The inflammatory response to malaria, both acute and chronic, follows a predictable sequence of events. Initial vascular changes precede increases in permeability, which ultimately causes oedema. Enhanced cytoadherence results in the accumulation, adherence, and migration of leukocytes through vascular endothelium. Molecular mediators are subsequently released and contribute to both the immune response and recruitment/activation of effector cells. Overwhelming evidence demonstrates that the pathophysiology of malaria involves both systemic and local cytokine release. The recruitment of phagocytes around cerebral capillaries has been observed in CM and likely explains increased chemotaxis and chemokinesis [31]. CM is a severe complication of *P. falciparum *infection that is characterized by cytoadherence in cerebral microvasculature. Accumulation of Hz-loaded monocytes has been observed in brains of CM victims [32] and may contribute to the disruption of endothelial basement membrane and subsequent extravasation of blood cells [26]. Importantly, blood brain barrier (BBB) destruction and enhanced vascular permeability/oedema are major factors associated with CM [33,34].

A potential contribution of 15-HETE toward increased vascular permeability has been examined in the lung. Administration of this hydroxylated fatty acid was shown to increase respiratory oedema fluid production [35], suggesting a role as an inflammatory mediator. The current analysis identified the "Leukocyte Extravasation Signaling" pathway as being significantly (*p *= 0.015) affected by 15-HETE. Specifically, the steady-state expression of *Arhgap12*, *Cldn11*, *Crkl*, *Mapk14*, and *Ptk2b *was up-regulated. Although 15-HETE is generally considered to have anti-inflammatory properties, activation of a large group of genes encoding inflammatory response molecules was observed (Table 2).

### 15-HETE and MMP9 Regulation

15-HETE was recently shown to enhance IL1β expression and MMP9 activity in human monocytes [26]. The current study identified a different response to 15-HETE. While *Il1b *mRNA was elevated in 15-HETE treated LPS-stimulated RAW 264.7 cells, *Mmp9 *mRNA was down-regulated (-4.8-fold by qRT-PCR). *Mmp9 *expression can be regulated through a variety of signaling cascades including NF-κB, p38 MAPK, and ERK1/2 pathways [36]. It was proposed that enhanced regulation of IL1B and MMP9 by 15-HETE in human monocytes may be associated with NF-κB signaling [26] based on reports demonstrating NF-κB-mediated MMP9 expression in LPS-stimulated RAW 264.7 cells [36]. This mechanism seems unlikely given that 15-HETE has been shown to impair NF-κB-mediated expression of *iNOS *in LPS-stimulated RAW 264.7 cells [13]. Furthermore, PPARγ ligands have been shown to repress NF-κB signal transduction [37,38] and inhibit *MMP9 *expression, secretion, and activity in macrophages and vascular smooth muscle cells [39-41], in accord with the results of this study.

## Conclusion

The complex innate and adaptive host immune responses to malaria are triggered by the presence of *P. falciparum *parasites, composite native Hz, Hz-derived lipid peroxidation products, and other cellular debris. A reductionist examination of the cellular response to two individual constituents of native Hz, BH and HNE [12], implicated a significant role for lipid peroxidation products in macrophage immuno-modulation. Consequently, the global response of macrophage-like cells to 15-HETE was explored. 15-HETE has previously been implicated as having a functional role in a variety of cellular processes such as inflammation, asthma, carcinogenesis, and atherosclerosis.15-HETE can be incorporated into membrane lipids and alter both vascular tone and EC permeability [14], supporting a potential role in malaria pathogenesis as well. In the current study, the response to 15-HETE was significantly associated with altered expression of "lipid metabolism" and "small molecule biochemistry" genes. Several genes related to "cytoadherence", "leukocyte extravasation", and "inflammatory response" were also differentially regulated by 15-HETE treatment. While these change uphold a potential role for 15-HETE in malaria pathogenesis, the small number of expression changes indicates that 15-HETE does not elicit a major response from macrophage-like cells in this model. These results add insight and detail to 15-HETE's effect on gene expression in macrophage-like cells, but there are limitations to any model system. For example, 15-HETE represents but one structural HETE isomer that is associated with Hz; 5-, 8-, 9-, 11-, and 12-HETE have also been identified [13,42]. 5- and 15-HETE are reported to be the predominant isomers formed during iron catalyzed or Hz-mediated oxidation of AA [42,43], yet 12-HETE may exert greater biological activity [13]. Current findings suggest that upon phagocytosis, the sum of all species adsorbed to the surface of Hz likely mediate a synergistic immuno-modulatory response. Future studies will address expression changes in primary human monocytes and macrophages in response to native Hz and Hz-associated lipid peroxidation products.

## Authors' contributions

ACS performed all analyses. ACS and DWW designed the study, wrote and approved the final manuscript.
